# Health and Quality of Life Perception in Older Adults: The Joint Role of Cognitive Efficiency and Functional Mobility

**DOI:** 10.3390/ijerph120911328

**Published:** 2015-09-10

**Authors:** Roberta Forte, Colin A.G. Boreham, Giuseppe De Vito, Caterina Pesce

**Affiliations:** 1 Institute for Sport and Health, University College Dublin, Dublin 4, Ireland; E-Mails: colin.boreham@ucd.ie (C.A.G.B.); giuseppe.devito@ucd.ie (G.D.V.); 2 Human and Health Sciences, Department of Movement, University of Rome “Foro Italico”, Roma 00135, Italy; E-Mail: caterina.pesce@uniroma4.it

**Keywords:** aging, cognition, executive function, walking, dual tasking

## Abstract

Cognitive and mobility functions are involved in health-related quality of life (HRQoL). The present cross-sectional study aimed at investigating what facets of efficient cognition and functional mobility interactively contribute to mental and physical HRQoL. Fifty-six healthy older individuals (aged 65–75 years) were evaluated for mental and physical HRQoL, core cognitive executive functions (inhibition, working memory, and cognitive flexibility), and functional mobility (walking) under single and dual task conditions. Multiple regression analyses were run to verify which core executive functions predicted mental and physical HRQoL and whether the ability to perform complex (dual) walking tasks moderated such association. Inhibitory efficiency and the ability to perform physical-mental dual tasks interactively predicted mental HRQoL, whereas cognitive flexibility and the ability to perform physical dual tasks interactively predicted physical HRQoL. Different core executive functions seem relevant for mental and physical HRQoL. Executive function efficiency seems to translate into HRQoL perception when coupled with tangible experience of the ability to walk under dual task conditions that mirror everyday life demands. Implications of these results for supporting the perception of mental and physical quality of life at advanced age are discussed, suggesting the usefulness of multicomponent interventions and environments conducive to walking that jointly aid successful cognitive aging and functional mobility.

## 1. Introduction

The worldwide rectangularization of the life expectancy curve with a huge rise in the proportion of the population that reaches old ages [1] urges societies to an increased understanding of how to ensure health and quality of life in older adulthood. Health-related quality of life (HRQoL) is a multi-dimensional construct reflecting the perception of the individuals’ state of health and ability to function in relation to the environment they live in [1,2]. Current research aims to describe both the HRQoL of older people and its determinants. This evidence base is useful to inform policy development for promoting intervention strategies and environmental conditions that lead to satisfactory levels of HRQoL perception at older age.

In general, good levels of HRQoL, emotional well-being, and life satisfaction depend on factors broadly ranging from socio-economic status to overall health, self-esteem, and the ability to maintain an active and independent lifestyle [3,4,5,6]. Executive cognitive function has also been found associated with HRQoL [7]. Executive function is an umbrella term for higher-level cognition involved in complex goal directed actions and behavioral adaptability [8]. Three core executive functions have been identified: the ability to inhibit mental routines and prepotent responses and control interference (inhibition), the ability to manipulate and update information held in working memory (working memory updating), and the ability to shift between mental sets (cognitive flexibility) [9]. Davis and colleagues [7] found both working memory and cognitive flexibility explaining a small, but significant, percentage of HRQoL variance. However, the authors used an instrument yielding a single summary score that reflects the broad construct of HRQoL. Indeed, debate is still ongoing on the use of specific or generic indicators [10,11]. Given the multifaceted nature of HRQoL, a more explanatory model of the relationship between the cognitive efficiency of older adults and their perception of health and quality of life might emerge if considering the specific contribution of executive functions to HRQoL distinguishing its mental and physical domains.

The efficiency of executive function is not only a dimension of mental health [12] that may translate in HRQoL perception in the mental domain; it may also contribute to HRQoL in the physical domain. In fact it allows a flexible allocation of cognitive resources when individuals have to cope with functional ability tasks highly relevant for independent living, as functional mobility; that is, efficient walking under various conditions typical of daily life (for review [13]).

The first aim of the present study was to evaluate the role of each of the three core executive functions in determining quality of life perceptions in the mental and physical health domains at advanced age. Cognitive flexibility seems not only associated with general HRQoL [7], but also with functional mobility [14,15]. Therefore, it may influence quality of life perception in the physical domain. Inhibition has been shown associated with functional mobility and efficiency in daily life activities too [14,16], but also with the emotional dimension of mental health [17]. Thus, we hypothesized that it might be related to HRQoL in both the physical and the mental domain. Regarding working memory, to our knowledge there is no evidence of a direct association with HRQoL except for that reported by Davis *et al.* [7], in terms of general HRQoL. One study evaluating both working memory efficiency and quality of life perception in different domains in older individuals did not measure their association, but their independent and different contribution to functional status [18].

The fact that executive functions were found explaining only a small percentage of HRQoL variance may not be exclusively due to the limited discrimination power of a global measure of HRQoL not differentiating across life domains [7]. It could be the case that subjective perception of HRQoL at advanced age may not be uniquely associated with diagnostic assessment outcomes, such as measures of cognitive efficiency. Rather, the extent to which older individuals perceive health and quality of life may also be influenced by tangible experiences related to mobility tasks common to everyday activities [19].

Indeed, functional status and life-space mobility seem to play a role in HRQoL perceptions not merely in the physical, but also in the mental domain [5,20]. Aging studies addressing the mobility-HRQoL relationship are mainly targeted to understand to what extent limitations of mobility due to conditions as obesity [21], dementia [22], critical illness or postoperative status [23,24] translate into poor HRQoL. However, functional mobility may be relevant to HRQoL not only when its physical prerequisites, as muscle strength and balance, are worsened by age-related adverse conditions [22]. Functional mobility is a multifaceted motor-cognitive competence, which tangible influence on daily life activities may render it a suitable factor moderating the relationship between executive function and HRQoL. Nevertheless, the joint role of efficient cognition and locomotion in predicting HRQoL perceptions of older people in the mental and physical domains had not been investigated yet.

Such an interactionist approach is relevant from an applied perspective on cognitive determinants to promote HRQoL in aging. In the field of interventional exercise and cognition research, growing interest is devoted to multi-component approaches that combine exercising executive function and functional mobility into integrated forms of locomotor dual task training with different levels of generality/specificity [25,26] (for review [27]). Such evidence shows promising effects not only on dual tasking ability [27], but also on executive function efficiency [15,26]. However, to tailor dual motor-cognitive training that are supportive of good quality of life in old age, we need to further our understanding of whether the ability of dual-task walking that is exercised in such types of motor-cognitive training supports the translation of executive function efficiency into good HRQoL perception.

Thus, we explored, secondly, whether the strength of the relationship between diagnostic measures of core executive functions and subjective perceptions of HRQoL is influenced by performance measures reflecting the tangible ability to cope with tasks that challenge functional mobility. Specifically, we tested whether the ability of older individuals to perform complex mobility tasks that involve mental effort moderates the relationship between executive functions and HRQoL perceptions. With advancing age, older adults must cope with increasing demands on mental resources to maintain efficient functional mobility, especially in complex conditions, such as when simultaneously controlling walking and other actions (*i.e.*, dual-task) [28].We hypothesized that it is not cognitive efficiency *per se* to help older people experience a good HRQoL. Instead the association may exist or be more pronounced when older individuals are able to exploit cognitive efficiency for mastering complex dual-task walking common in daily life.

## 2. Experimental Section

This is a cross-sectional study of the relationship between executive function and mental/physical HRQoL and of the potential moderation by functional mobility in healthy older individuals.

### 2.1. Recruitment and Participants

Information leaflets with details about the study were sent to individuals of both genders, aged 65–75 years, who had expressed interest in the project as advertised in local university and parish newsletters. Of the 65 wishing to take part, based on activity level and health history, 56 met the selection criteria. To exclude confounding effects of health status on functional performance, cognition, and HRQoL, inclusion criteria entailed: absence of musculoskeletal or neurological diseases or of severe arthritis, cardiac illness, history of cerebro-vascular disease, or uncontrolled metabolic disease [29]. Additionally, to exclude any influence of individual differences in fitness status, the selection criterion was regularly exercising less than twice a week, as exercise guidelines for older individuals prescribe ≥2–5weekly sessions of exercise to induce significant fitness changes [30].

Ethical approval was obtained from the ethics committee of the local University (project identification code: Re: LS-09-78-Forte-Boreham, date of approval: 6 July 2009), and informed consent was signed by each participant.

### 2.2. Testing

First, body mass, stature and body composition through dexa scan were measured. The latter was used to obtain an index of sarcopenia (*i.e.*, sum of appendicular skeletal muscle mass in kg/height^2^ [31]); that is, a deficiency in relative muscle mass associated with difficulties in functional performance and daily life activities. Thereafter, participants were tested for core executive functions (Random Number Generation task (RNG), [32]; Trail Making test, [33]), functional mobility [14,25], and HRQoL [34]). The Random Number Generation task (RNG) was used to measure inhibition and working memory updating, whereas the Trail Making test was used to test cognitive flexibility.

The RNG task requires participants to verbally generate a random sequence of numbers included between one and nine in time with a metronome set at 40 bpm (*i.e.*, with an inter-beat interval of 1.5 s). Prior to data collection by tape recording, participants performed a familiarization trial. Both the omission of a number generation in correspondence of one tone and the production of numbers lower than one (zero) or higher than nine (10, 11, *etc.*) were considered as errors and discarded. If errors exceeded a predefined maximum threshold of five, the entire block was repeated. Eighteen different indices of randomness were computed [32]. Six indices were selected, as they reflect two of the three core executive functions of interest: inhibition of mental routines (Turning Point Index (TPI), Adjacency score (Adj) and Runs score (Runs)) and working memory updating (Redundancy score (Red), Coupon score (Coupon), and Mean Repetition gap (MeanRG)) [32,35]; for a detailed explanation of indices computation see [36].

Briefly, the TPI is a measure of the similarity between the real frequency of turning points, marking a change between ascending and descending series of numbers (e.g., the response change between the digits “2” and “5” in a hypothetical sequence “9, 7, 2, 5, 6, 8”) generated by the participant and their theoretical frequency in random responses. A TPI lower than the optimal value of 100 indicates that participants produced more or fewer turning points than theoretically expected. The Adj measures the relative frequency of pairs of adjacent ascending or descending numbers (e.g., 7–8 or 4–3) as compared to the total number of response pairs produced by the participant. It ranges between 0% and 100% and The Runs is an index of variability of the number of digits in successive ascending or descending runs. Variability is highest and leads to lowers cores when the participant alternates ascending and descending pairs of digits as “4, 7, 9, 2”.

The Red index reflects the imbalance of response alternative frequencies in the sequence that derives from a more frequent usage of given numbers than expected based on the theoretical frequency in random responses. Repeating the same digit along the whole sequence would lead to a Red score of 100% (complete redundancy). The Coupon score measures the mean number of digits generated until the entire set of alternatives has been used. If the participant omits to generate one of the available digits throughout the sequence, the Coupon is the highest that is equal to the number of digits composing the sequence (*i.e.*, 100). The MeanRG is the mean number of responses given until each digit reoccurs, calculated for all digits throughout the whole sequence (e.g., in the sequence “2, 8, 4, 6, 2, 9, 7, 8”, the digits “2” and “8” reoccur with a mean gap equal to 4). Repeating one or more items much more frequently than theoretically expected leads to a low MeanRG value.

TPI, Adj, and Runs were merged into an average index of inhibition and Red, Coupon, and MeanRG into an average index of memory updating. High levels of Turning Point Index, but low values of Adj and Runs, reflect a good ability to suppress the habitual tendency to count forward or backward, as well as high levels of MeanRG, but low levels of Red and Coupon reflect a good ability to update information on already generated or still not generated digits held in working memory. Thus before averaging, all indices were *z* standardized and Adj, Runs, Red, and Coupon were reversed [25].

The trail making test is a test of attention, speed, and mental flexibility [33]. For this test the participant is required to make a trail with a pencil joining 25 circles distributed over a sheet of paper. The test is composed of two parts (A and B). In part A the circles are numbered 1 to 25; in part B the circles include numbers (1 to 12) and letters (A to L). For both parts participants are asked to draw lines to connect the circles in an ascending order, numbers only for part A and alternating between the numbers and letters in part B (*i.e.*, 1-A-2-B-3-C *etc.*). The test was demonstrated to participants on a sample sheet, then a copy of the test worksheet and a pencil was given. They were instructed to connect the circles as quickly as possible, without lifting the pencil from the paper. The start number/letter and the finishing number/letter were shown and time recording in seconds started when participants initiated the trail and stopped when the last number/letter was connected. As correction of errors forms part of the completion time for the task, any errors were pointed out immediately to participants by reminding them of the correct sequence (*i.e.*, number, or letter). They were then asked to revert to the last correct number/letter reached and continue from there.

Health and quality of life was assessed using the SF-36^®^ questionnaire version 2 [34]. This questionnaire comprised 36 questions regarding behavioural functioning, perceived well-being, social, and role disability and perception of health in general. Scores were coded, summed, and transformed onto a scale from zero (worst possible health) to 100 (best possible health) using the method described by the manufacturer. Summary measures of mental (MCS) and physical (PCS) health were calculated as prescribed [34] and used for the purpose of the study.

Functional mobility was assessed as maximal walking speed (WS; m/s), *i.e.*, walking as fast as possible without running, over 10 meters in simple task and in two dual-task conditions [14,37]:

(1) Motor-motor dual-task: participants walking at maximal speed stepped with their preferred foot over two plastic hurdles of 15 and 45 cm height placed in succession on the mid-line of the course (hurdles WS).
(2) Motor-cognitive dual-task: participants were asked to talk while walking, naming as many animals as they could beginning with either letter B or C (talk WS).

Maximal and not habitual walking speed was chosen to generate time pressure that rendered the mobility task more challenging and presumably more strongly relying on executive function. The dual task conditions under time pressure, either being walking and talking or walking and responding to changes in the walking terrain (*i.e.*, negotiating obstacles), were chosen because such capability represents a critical requirement for mobility which, if not managed successfully, can lead to mobility disability and loss of independency in activities of daily life [38].

### 2.3. Statistical Analysis

Initial analyses included calculation of descriptive statistics and of Pearson’s correlation coefficient on all variables of interest (HRQoL, executive function, dual-task functional mobility) and variables to be controlled for potential covariation (index of sarcopenia, maximal walking speed). Independent samples *t*-tests were also performed to preliminarily test for gender differences in all variables.

The first question of this cross-sectional study regarded the role of executive functions in determining HRQoL separately in the mental and physical health domains. To answer this question, two exploratory multiple regression analyses were performed to test the prediction of MCS and PCS accrued by the three core executive functions: inhibition, working memory, and cognitive flexibility. The three executive function variables were entered simultaneously in the multiple regression model after a preliminary bivariate correlation analysis (Pearson’s r) to ensure the absence of multicollinearity.

The second question regarded whether the contribution of executive functions to HRQoL is influenced by the ability of older people to successfully perform functional mobility tasks that rely on executive functions. To answer this question, subsequent analyses were run that tested the moderating role of functional mobility on executive functions in predicting HRQoL. Due to the limited sample size, we did not run a comprehensive regression model including all executive function and functional mobility variables and their interaction terms, but run three regression models separately testing whether each core executive function was predictive of HRQoL under specific functional mobility conditions. Thus, MCS and PCS were separately regressed on each core executive function (inhibition, working memory updating, or cognitive flexibility) with dual-task functional mobility performances (hurdles WS and talk WS) as moderators. Before running the moderated prediction models, a preliminary bivariate correlation analysis (Pearson’s r) between executive functions and dual-task WSs was run to exclude associations between predictor and moderator variables. Index of sarcopenia and maximal walking speed were included in each moderated prediction model as covariates. The rationale for including these covariates was that we aimed at disentangling the role played by the ability to walk under dual tasking conditions that mirror everyday life challenges from their neuromuscular prerequisites. In this way, we aimed at limiting spurious results, as neuromuscular fitness is both a determinant of functional mobility [39] and a factor influencing cognitive efficiency [25].

The analyses used to test moderated prediction entailed the following steps.

(1) Interaction variables were computed by multiplying each executive function by each functional mobility variable.
(2) Three separate hierarchical multiple regression analyses with forced entry method were run for the prediction of MCS and PCS, one for each main executive function predictor. Maximal walking speed and index of sarcopenia were statistically controlled for by entering them in a first block, while the individual predictors (one executive function variable and the two dual-task WS variables) were entered in a second block and the interaction terms of the executive function with the dual-task WSs in a third block.
(3) In case interaction terms significantly predicted MCS or PCS, *posthoc* analysis through a simple slope test was performed [40]. The statistical significance was set at *p* < 0.05.

## 3. Results

### 3.1. Descriptive Statistics and Preliminary Analyses

Descriptive statistics and results of the *t-*tests performed to preliminarily control for gender differences are reported in Table 1. Gender differences were observed in height, weight, and % body fat, as well as some of the dependent and independent variables: index of sarcopenia and walking speed negotiating hurdles in favor of men.

**Table 1:** Mean ± standard deviation of all variables measured in all participants (n = 56). Significant differences between males and females are also reported.

	All	Men	Women	*p*
Age (years)	69.6 ± 3.2	70.0 ± 3.3	69.4 ± 3.2	n.s.
Height (cm)	1.7 ± 0.1	172.9 ± 5.0	162.4 ± 7.0	<0.001
Weight (kg)	72.8 ± 11.5	81.2 ± 9.6	66.7 ± 8.4	<0.001
Body fat (%)	33.8 ± 7.9	28.7 ± 5.2	37.5 ± 7.4	<0.001
Sarcopenia (index)	7.6 ± 1.3	8.8 ± 0.8	6.8 ± 0.8	<0.001
Maximal WS (m/s)	1.9 ± 0.2	1.91 ± 0.2	1.8 ± 0.2	n.s.
Talking WS (m/s)	1.6 ± 0.3	1.6 ± 0.3	1.6 ± 0.2	n.s.
Hurdles WS (m/s)	1.6 ± 0.2	1.7 ± 0.2	1.6 ± 0.2	<0.001
Inhibition (std index)	0.098 ± 0.8	−0.08 ± 0.9	0.05 ± 1.0	n.s.
Working memory (std index)	−0.013 ± 0.8	−0.33 ± 1.07	0.22 ± 0.5	0.020
Δ Trail (s)	43.0 ± 29.1	52.9 ± 34.7	39.1 ± 29.5	n.s.
MCS (score)	83.8 ± 13.2	84.1 ± 12.6	82.9 ± 14.3	n.s.
PCS (score)	71.0 ± 13.4	71.4 ± 15.4	70.1 ± 12.3	n.s.

Notes: MCS = mental HRQoL component; PCS = physical HRQoL component.

For what concerns the analysis of the relationship among HRQoL and the cognitive and mobility variables, calculation of bivariate correlation coefficients (Pearson’s r) did not reveal any significant association with the exception of that between inhibition and mental HRQoL (Table 2).

**Table 2:** Results of correlation analysis (Pearson’s r) between functional mobility (WS = walking speed) and executive function (Inhibition, Working memory, Cognitive flexibility (Δ Trail)) measures and the mental (MCS) and physical (PCS) components of HRQoL.

	Maximal WS	Hurdles WS	Talking WS	Inhibition	Working Memory	Δ Trail
MCS	−0.057	0.178	−0.082	0.359**	−0.030	−0.063
PCS	−0.083	0.103	−0.114	0.113	−0.111	−0.112

Note: ** *p* = 0.007.

Correlational analyses did not reveal any collinearity among executive function variables that might affect the outcomes of the exploratory prediction model, or between executive function and functional mobility variables that might affect the outcomes of the moderated prediction model. The correlation between inhibition and working memory was negligible (r < 0.1) and that between cognitive flexibility and inhibition or working memory was significant, but weak to moderate (r < 0.4). Correlations between executive function and functional mobility variables were negligible (<0.15) and non-significant.

### 3.2. Moderated Prediction Analyses

The exploratory multiple regression analyses performed to test the prediction of MCS and PCS accrued by executive function variables showed that inhibition was the only significant predictor of MCS. The model of the PCS did not reveal any significant predictor (Table 3). VIF statistics (<2) indicated the absence of multicollinearities.

**Table 3:** Multiple regression models for the prediction of mental (MCS) and physical (PCS) HRQoL accrued by core executive functions (inhibition, working memory, and cognitive flexibility). Total R^2^ explained, ANOVA results and standardized β coefficients with the level of significance are also reported.

**Exploratory** **Prediction Model**	**PCS**	**R^2^ = 0.14**	**F_3,52_ = 2.78, p = 0.050**
		β	p
	Inhibition	0.337	0.016
	Working Memory	−0.084	0.548
	Cognitive Flexibility	−0.092	0.528
	**PCS**	**R^2^ = 0.08**	**F_3,52_ = 1.49, p = 0.230**
		β	p
	Inhibition	0.029	0.834
	Working Memory	−0.205	0.160
	Cognitive Flexibility	−0.271	0.077

The results of the moderated regression analyses performed to answer the second question on whether the contribution of executive functions to HRQoL is influenced by the ability of older people to successfully perform functional mobility tasks are reported in Table 4. Only one regression model for each HRQoL variable (MCS, PCS) yielded significant results: the interaction inhibition × talking while walking predicted MCS (*p* < 0.05) and the interaction Δ Trail × hurdle walking predicted PCS *p* < 0.05). VIF statistics (<2) indicated the absence of multicollinearities.

**Table 4:** Hierarchical regression models testing moderated prediction of mental (MCS) and physical (PCS) HRQoL. Total R^2^ explained, ANOVA results, and standardized β coefficients with level of significance are also reported. (WS = walking speed; Δ Trail = cognitive flexibility).

**Moderated Prediction Model**	**MCS**	**R^2^ = 0.25**	**F_5,54_ = 3.25, p = 0.013**
		β	p
	Maximal WS	−0.171	0.302
	Index of Sarcopenia	−0.169	0.197
	Inhibition	0.211	0.133
	Talking WS	−0.119	0.476
	Talking WS × Inhibition	0.343	0.031
	**PCS**	**R^2^ = 0.18**	**F_5,55_ = 2.17, p = 0.72**
		β	p
	Maximal WS	−0.350	0.039
	Index of Sarcopenia	−0.048	0.723
	Cognitive Flexibility (Δ Trail)	−0.041	0.728
	Hurdles WS	0.325	0.053
	Hurdles WS × Δ Trail	−0.360	0.020

**Figure 1:**
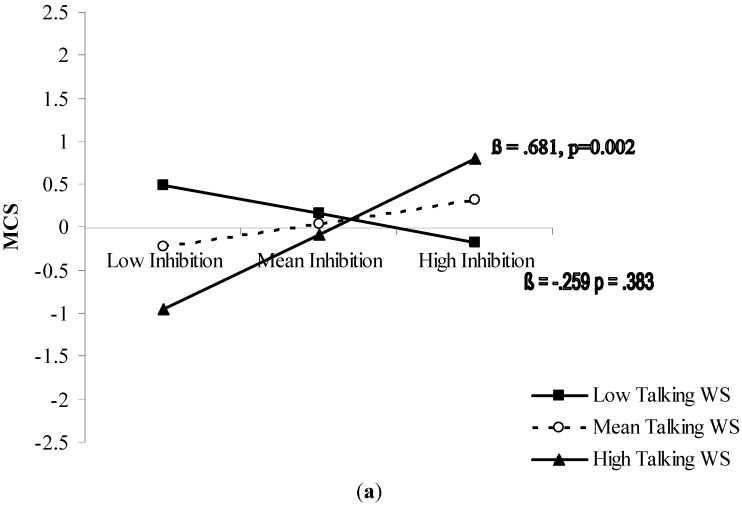

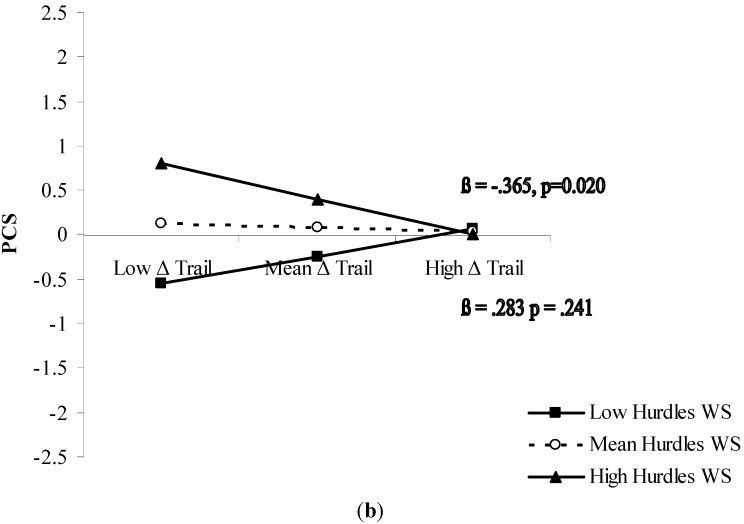
(**a**) Prediction of MCS accrued by inhibition capacity and moderated by walking speed while talking; and (**b**) prediction of PCS accrued by mental flexibility (good mental flexibility = low Δ Trail) and moderated by walking speed while negotiating hurdles. Solid lines: change in the slope of the predictor for high *vs.* low levels (1 sd change) of the respective moderator; value of (β) and its significance are reported. (WS = walking speed).

The significant interactions were further analyzed with simple slope testing (Figure 1). In both cases, the moderation analysis revealed an amplifying effect of the moderator on the predictor. For the MCS (Figure 1a), inhibition was a significant predictor (*p* < 0.01) of the mental component of HRQoL when accompanied by good ability in walking while talking. As concerns PCS (Figure 1b), results showed that cognitive flexibility determined good perception of the physical component of HRQoL (*p* < 0.05) when was accompanied by good ability to walk while negotiating hurdles.

## 4. Discussion

Increasing recognition of the importance of both cognitive and physical functioning in determining how older people feel quality of life makes it necessary to further our understanding of this relationship. An interactionist approach represents an attempt to describe the complete picture for applied purposes. In sum, the present study showed a differentiated pattern of association between executive cognitive function and HRQoL perception in the mental and physical domains. Core executive functions results were differently associated with the mental and physical components of HRQoL. Inhibitory ability seems relevant for mental HRQoL (Figure 1a), whereas cognitive flexibility seems relevant for physical HRQoL (Figure 1b). Both relationships have in common an amplifying moderation by functional mobility.

A large amount of research has proven that good functional ability is relevant to perceive good health and quality of life, well-being, and life satisfaction [5,6,19,41] at older age, while only a few studies have evaluated the importance of cognitive measures of mental health for HRQoL perception [42]. This imbalance is not surprising, since clinical measures of health status alone do not determine better quality of life [43]. For older individuals to perceive life satisfaction it seems more meaningful to be efficient in daily tasks involving functional mobility [19]. However, on the other hand, improvements in functional ability does not necessarily bring gains in general well-being [44] and specific mental health [45], thus suggesting a lack of direct association.

The joint contribution of executive function and functional mobility to HRQoL perception at older age is a novel result suggesting that functional mobility acts as a moderator of the relationship between executive function efficiency and HRQoL perception of older people. Only when coupled with tangible experience of ability to cope with complex functional mobility tasks of daily life, executive function efficiency seems predictive of feeling good health and quality of life.

The present findings allow differentiating what type of executive functions and ability to navigate within complex environs may jointly translate into good mental or physical HRQoL perceptions. It appears that the role played by experiences of successful locomotor dual tasking is domain-specific. Motor-cognitive dual tasking, e.g., talking while walking, and motor-motor dual tasking, e.g., walking while negotiating hurdles, seem to be involved in quality of life perception in the mental domain and in the physical domain, respectively.

Older individuals who are able to effectively inhibit mental routines do not necessarily perceive good mental health. Only those who presumably capitalize on their inhibitory ability to talk while walking do (Figure 1a). A similar pattern was observed as concerns the joint role of cognitive flexibility and complex walking ability in determining physical health feelings: older individuals having good cognitive flexibility do not necessarily feel good physical health. Only those who seem able to exploit their cognitive flexibility for efficient walking while negotiating hurdles do (Figure 1b).

During mobility tasks, efficient executive function makes it possible to appropriately control limb coordination to generate gait [13]. In particular, inhibition may play a key role in suppressing dysfunctional co-movements during coordinated locomotor actions [14]. Furthermore, at an older age, inhibition is related to the efficiency of the “supervisory attention system” that modulates attentional allocation to different tasks as in dual-task conditions [46]. Our results add to this evidence, suggesting that, to experience mental well-being, older individuals need efficient inhibition and, capitalizing on it, good ability to deal with critical dual tasks common to social activities such as talking while walking.

The literature generally shows that being cognitively flexible is a requisite of complex functional ability [16]. Our results suggest that being cognitively flexible is a necessary, but not sufficient requisite to experience physical well-being at older age. Cognitive flexibility translates into feeling good physical health only when older individuals are also able to use this flexibility to walk while negotiating obstacles, commonly needed to navigate within complex environs to successfully reach the desired location.

One of the three core executive functions, working memory updating, was unexpectedly not associated with HRQoL in either tested domains. This is in disagreement with the finding by Davis *et al.* [7]. The reason may not only be the unspecific nature of Davis *et al.*’s summary measure of HRQoL, but also to the multifaceted nature of working memory that consists of three main components: the phonological loop, the visuospatial sketch pad, and the central executive. Davis *et al.* [7] used the verbal digit span test (difference between backward and forward test scores) that represents a measure of the central executive component of working memory. Additionally, the three indices of working memory updating of the RNG task employed in the present study mainly tap the central executive component of updating [35]. However in random generation, more dynamic memory tracking or updating is challenged, as there is the need to maintain representations of responses as well as integrate previous with current choices, selecting among competing response candidates over time. This specific and highly challenging updating requirement may not be strictly related to everyday working memory requirements on which older people build their mental quality of life perception.

The above interpretations of how specific core executive functions interact with specific dual-task walking abilities in determining quality of life perceptions in the mental and physical domains are merely speculative. However, the coherent, domain-specific association between functional mobility moderators and quality of life perceptions (*i.e.*, motor-cognitive dual-tasking ability with mental HRQoL and motor-motor dual-tasking ability with physical HRQoL) limits the risk that our speculations are arbitrary and indicates the usefulness of an interactionist approach to inform interventional design development.

The main limitations of the present study include the cross-sectional nature of the study and the small number of participants. The inclusion of only older adults and the unavailability of longitudinal data represent a limitation, as aging studies have indicated the risk for underestimation when using cross-sectional regression models as compared to longitudinal models [20]. Moreover, this study was not powered formally, as it was intended to produce data necessary to adequately power a full scale study. Due to the limited sample size (n = 56), to test moderated prediction we did not run a comprehensive regression model including all executive function and functional mobility variables and their interaction terms, but run three regression models separately testing the moderated prediction by each core executive function. Thus, the possibility of spurious findings represents a major limitation. However, the inclusion of appropriate covariates allowed disentangling the role of executive function and locomotor dual-tasking from neuromuscular fitness factors.

While low levels of regular physical activity are representative of the lifestyle habits of the majority of older adults in the considered age range on the island of Ireland [47,48], a limitation of the study is represented by the homogeneous healthy conditions of the low active participants, which make results not extendable to the general aging population. Participants of the present study were healthy as reflected in their perceived mental and physical health, similar to those of individuals of the same nationality [49]. Their good health status was confirmed by functional mobility data, walking speed performances being comparable to reference data reported for healthy aging individuals [50]. Moreover, the HRQoL and executive functions tests though standardized and widely used may not represent a comprehensive assessment. Nevertheless, the study attempts to make a step forward in the study of the diversity and interrelation of personal abilities that allow older individuals feel a good health and quality of life.

## 5. Conclusions

From a practical point of view, the present findings represent valuable information to be used when planning interventions targeted to preserve or improve HRQoL of older adults in both mental and physical domains. Cognitive training practices have been successfully demonstrated for limiting age-related decline in executive function [51]. However, the present results suggest that for executive function efficiency to translate into perception of good mental and physical health in older adulthood, it must be coupled with tangible experience of ability to cope with everyday life demands relying on executive function, such as walking under dual task conditions. Therefore, to ensure that older people perceive mental and physical quality of life, it might be insufficient to apply cognitive training practices targeted to executive functioning. Rather, it seems necessary to also train all the physical fitness and neuromuscular factors contributing to functional mobility. Multicomponent, mind-body practices that are recently receiving attention in aging research [25,26,27] seem best suitable to this aim. More broadly, it is to consider that not only structured physical activity interventions, but also an active lifestyle and particularly walking habits have a protective effect against both cognitive executive [52] and functional ability decline [53] in older adulthood. Thus, both specifically-designed physical activity interventions focused on dual task practices [27] and actions impacting the built environment to render it more conducive to physical activity [54], especially walking [55], may represent complementary means to catch two birds with one stone to jointly contribute to HRQoL perception in aging: executive function and functional mobility.
